# Pathway as a Pharmacological Target for Herbal Medicines: An Investigation from *Reduning Injection*

**DOI:** 10.1371/journal.pone.0123109

**Published:** 2015-04-01

**Authors:** Jianling Liu, Ke Sun, Chunli Zheng, Xuetong Chen, Wenjuan Zhang, Zhengzhong Wang, Piar Ali Shar, Wei Xiao, Yonghua Wang

**Affiliations:** 1 College of Life Science, Northwest University, Xi’an, Shaanxi, 710069, China; 2 College of Life Science, Northwest A & F University, Yangling, Shaanxi, 712100, China; 3 Center of Bioinformatics, Northwest A & F University, Yangling, Shaanxi, 712100, China; 4 State Key Laboratory of New-tech for Chinese Medicine Pharmaceutical Process, Lianyungang, Jiangsu, 222001, China; Royal College of Surgeons, IRELAND

## Abstract

As a rich natural resource for drug discovery, Traditional Chinese Medicine (TCM) plays an important role in complementary and alternative medical systems. TCM shows a daunting complexity of compounds featuring multi-components and multi-targets to cure diseases, which thus always makes it extremely difficult to systematically explain the molecular mechanisms adequately using routine methods. In the present work, to reveal the systematic mechanism of herbal formulae, we developed a pathway-based strategy by combining the pathways integrating, target selection, reverse drug targeting and network analysis together, and then exemplified it by *Reduning injection* (RDN), a clinically widely used herbal medicine injection, in combating inflammation. The anti-inflammatory effects exerted by the major ingredients of RDN at signaling pathways level were systematically investigated. More importantly, our predicted results were also experimentally validated. Our strategy provides a deep understanding of the pharmacological functions of herbal formulae from molecular to systematic level, which may lead to more successful applications of systems pharmacology for drug discovery and development.

## Introduction

Traditional Chinese Medicine (TCM) plays an important role in human health through thousands of years of clinical practice [1]. However, the molecular mechanisms involved in TCM still remain somewhat unclear. Herbal medicines are normally designed and featured as multicomponent systems that target multi-targets [2], which makes it hard to decipher the mechanisms of action like those single target drugs by traditional pharmacological methods.

Recently, systems pharmacology is increasingly gaining acceptance as a promising way to address the complex problems for herbal medicines [3]. In our previous work, we have successfully built an integrated platform of systems pharmacology and applied it into the discovery of bioactive ingredients [4], prediction of drug targets [5], exploration of therapeutic mechanisms [6,7] and reveal of TCM combination rule [8,9], etc. This platform mainly focus on a large-scale analysis to find the bioactive compounds and targets and then build drug-disease connections to reveal how herbal medicine actually works on diseases [6].

As we know, the protein targets contained in signaling pathways have been used in drug discovery, and several drugs targeting pathways have reached preclinical and clinical trials for a number of diseases, such as cancer and autoimmune disorders therapy [10–12]. But the signaling pathway-interacting networks are rarely highlighted in the existing processes for drug discovery. As a matter of fact, the protein-protein interactions, protein phosphorylation and protein-DNA interactions in signaling pathways often lead to several properties such as extended signal duration, activation of feedback loops and multiple inhibitions [13]. The pathway-based combinatorial therapies which modulate these interactions through a regulation of the target activities in multiple signaling pathways may become a necessity for the maximization of therapeutic efficacy [14]. Thus, the mechanistic studies of signaling pathway networks may offer further therapeutic opportunities for drug discovery and clinical applications.

In this work, we provided a systems-based method to explore the therapeutic mechanism of TCM for a given disease based on signaling pathways. As an example, we experimentally verified this method by *Reduning injection* (RDN) in combating inflammation [15]. This work attempts to offer a novel thinking for deep understanding of biological basis and pharmacology of herbal medicines, as well as a methodology for drug discovery and development.

## Materials and Methods

### *In slico* modeling and analysis

**Fig 1:**
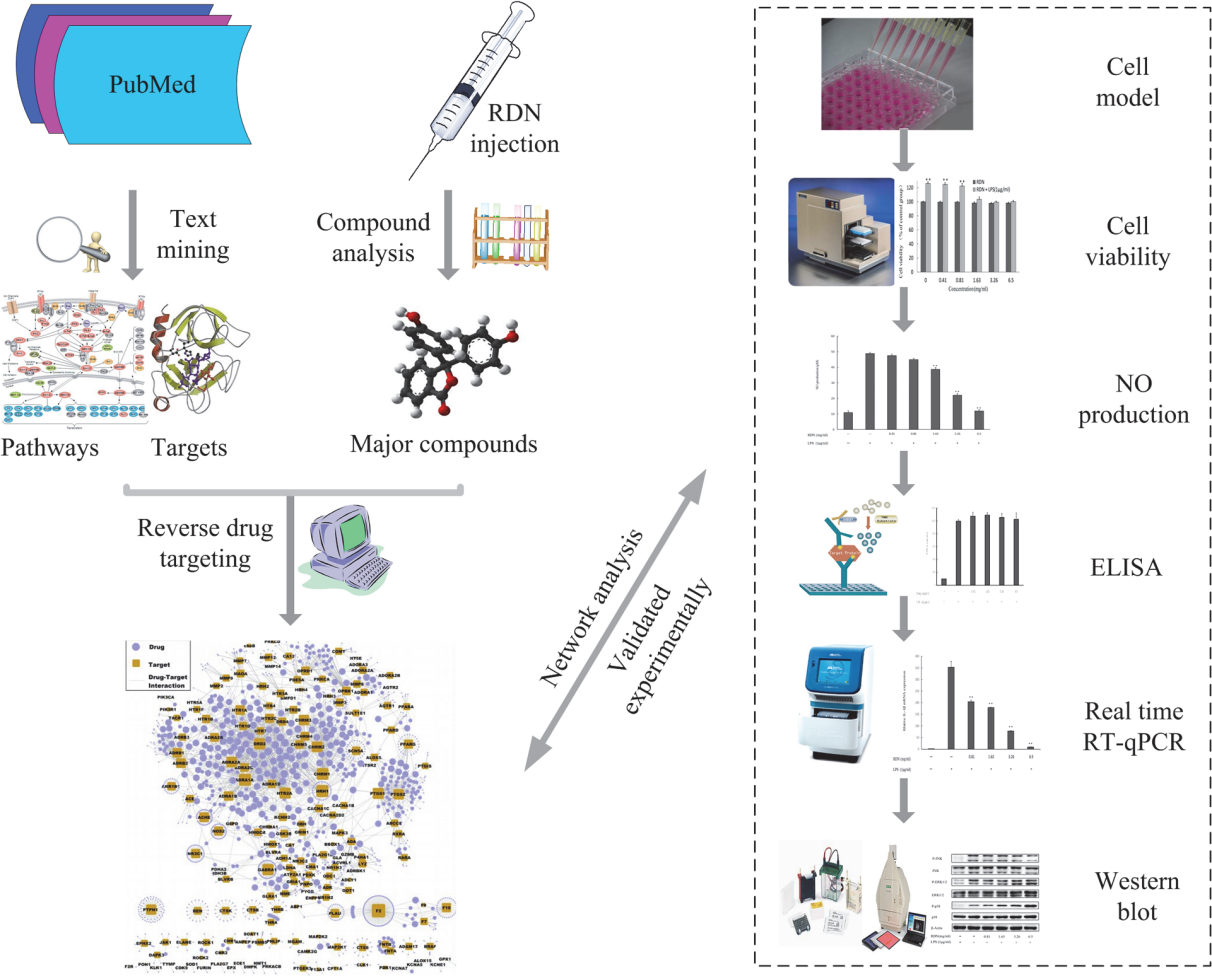
Workflow for signaling pathway-based strategy.

The signaling pathways-based approach integrates pathways integration, reverse drug targeting and network construction, and then topological analysis was applied to identify the potential therapeutic mechanism. The detailed descriptions of this tactic are shown in **Fig 1**.

#### Potential target identification

A large-scale text mining and manual check (from 1995 to 2013) with the keywords “inflammation” and “pathways” were performed to acquire the existing pathways that are well identified in the inflammation-associated diseases. Here, NF-κB (nuclear factor kappa B) and MAPKs (mitogen-activated protein kinases) pathways are the key players in the progression of inflammation [16–18], and the protein IκB (inhibitor of kappa B), p65 (transcription factor p65), ERK (extracellular signal-regulated protein kinases), JUN (c-Jun N-terminal kinases) and p38 (p38 mitogen-activated protein kinases) in these pathways are major or marker drug targets, together with the downstream proteins iNOS (inducible nitric oxide synthase), COX-2 (cyclooxygenase 2) and TNF-α (tumor necrosis factor α) [19,20]. The crystal structures of selected targets were fetched from the RCSB Protein Data Bank (http://www.pdb.org/pdb/home/home.do). The PDB IDs of these target proteins are as follows: IKBKB: 4KIK, RELA: 3QXY, MAPK 1: 1TVO, MAPK 3: 2ZOQ, MAPK 8: 4AWI, MAPK 9: 3NPC, MAPK 11: 3GC8, MAPK 12: 1CM8, MAPK 13: 4EYM, MAPK 14: 1OZ1, NOS 2: 3E7G, PTGS 2: 1CVU, TNF: 2AZ5.

#### Reverse drug targeting

*Reduning injection* is a clinically widely used herbal medicine injection in combating inflammation in China. RDN is composed of three herbs including *Artemisiae annuae L*. (genus *Artemisia*, Asteraceae), *Gardenia jasminoides J*.*Ellis* (genus *Gardenia*, Rubiaceae) and *Lonicera japonica Thunb*. (genus *Lonicera*, Caprifoliaceae) [15], which is a mixture of 75 chemicals including 16 iridoids, 14 lignans, 15 phenolic acids, 11 flavonoids, 12 caffeoylquinic acid derivatives, 4 sesquiterpenoids, 3 coumarin compounds, etc [21].

To probe the actual bioactive compounds in RDN and their targets, ten ingredients which are abundant (possess a relatively large concentration) in RDN were taken out from the whole 75 chemicals and their chemical structures were obtained from NCBI PubChem database (http://www.ncbi.nlm.nih.gov/pccompound) or the Chemical Book (http://www.chemicalbook.com) (**Table 1**). By using two mathematical tools of Random Forest (RF) and Support Vector Machine (SVM), the chemical, genomic and pharmacological information were efficiently integrated to predict the drug–target associations [22]. In this study, RF value ≥ 0.7 or SVM ≥ 0.8 were chosen as threshold to screen the potential candidate compounds.

**Table 1:** The chemical IDs, names and the inhibition ratio of NO production for ten relatively abundant compounds in RDN.

ID	Compound name	Concentration1	Concentration2	Concentration3
M1	Isochlorogenic acid A	1.04 ± 1.95	-1.18 ± 2.87	-1.07 ± 2.88
M2	Gardenoside	0.50 ± 1.89	-1.15 ± 1.72	-0.11 ± 2.53
M3	Geniposide	-1.12 ± 0.91	0.56 ± 2.13	1.09 ± 2.61
M4	Geniposidic acid	0.56 ± 1.88	-0.01 ± 2.5	0.47 ± 2.55
M5	Isochlorogenic acid C	-0.84 ± 2.69	-1.11 ± 1.77	-1.83 ± 2.09
M6	Chlorogenic acid	-1.21 ± 2.58	-5.72 ± 1.15	-6.43 ± 1.89
M7	Secoxyloganin	0.02 ± 2.34	-0.49 ± 1.62	-1.38 ± 0.88
M8	Genipin	75.97 ± 1.1	53.75 ± 2.61	26.71 ± 1.55
M9	Scopoletin	64.57 ± 1.82	28.25 ± 3.91	10.49 ± 1.26
M10	Salicylic acid	0.97 ± 2.30	-1.08 ± 1.13	-0.35 ± 1.35

RAW 264.7 cells were pre-treated with three 2-fold serial diluted concentrations of test samples named concentration 1, 2 and 3 for 2 h. After stimulated with LPS (1μg/ml) for 24 h, the inhibition ratio of NO production was calculated. Initial concentrations of agents used are: 100 μM for genipin, 400 μM for scopoletin and 800 μM for others. Data were presented as mean ± standard error of three independent experiments. P < 0.05 represents significant difference compared with the cells that treated with LPS only.

#### Network building and analysis

To make a deep understanding of the action mechanism of RDN, the drug-target (D-T) network was constructed by Cytoscape 2.8 [23] where the candidate compounds and their potential targets were connected if the protein is a predicted target of the compound, and the key topological parameter degree was analyzed. The degree of a node characters as the number of edges associated to it, indicating the importance of the node in a network.

### Experimental validation

#### Test sample preparation

RDN, chlorogenic acid, isochlorogenic acid A, isochlorogenic acid C, gardenoside, gardenoside and secoxyloganin were kindly provided by Jiangsu Kanion Pharmaceutical Co., Ltd (Lianyungang, Jiangsu, China). And geniposide, geniposidic acid, genipin, scopoletin and salicylic acid were purchased from Nanjing Zelang Medical Technology Co., Ltd (Nanjing, Jiangsu, China). Test samples, except for RDN injection, were dissolved in DMSO (Sigma, USA) to make a stock solution of 800 mM, and the final concentrations of DMSO presented in the culture media (less than 0.1%) had no effect on cell viability.

#### Cell culture

RAW 264.7 cells (Cell Bank of the Chinese Academy of Sciences, Shanghai, China) were cultured in 25 or 75 cm^2^ flasks with Dulbecco's modified Eagle's medium (DMEM) (Gibco BRL, USA) supplemented with 10% fetal bovine serum (FBS) (Gibco BRL, USA) at 37°C in 5% CO_2_/95% air.

#### Cell viability assay

RAW 264.7 cells at a density of 4×10^5^ cells/ml were seeded into 96-well plate and incubated for 24 h for cell accommodation, then the culture supernatant was replaced with 100 μl of fresh medium with or without various concentrations of test samples for 2 h and incubated for another 24 h with or without 1 μg/ml of LPS (E.Coli 055:B5) (Sigma, USA) under normal conditions. The effects of our test samples on cell viability were tested using CCK-8 assay (BestBio, Shanghai, China). To acquire accurate absorbance, the background of test samples were eliminated by removing whole culture medium and washing twice with preheated PBS, then 100 μl/well fresh medium with 10% CCK-8 solution was added, the OD values were read at the wavelength of 450 nm on an microplate reader (Molecular Devices, California, USA) after incubated at 37°C for 1 h.

#### Measurement of NO and cytokine production

To investigate the anti-inflammatory effect of the formula, NO and TNF-α production in LPS-stimulated RAW 264.7 cells were measured. RAW 264.7 cells (4×10^5^ cells/ml) seeded in 96-well plates were cultured for 24 h, after pre-treated with different concentrations of test samples for 2 h and subsequently treated for 24 h with LPS (1 μg/ml). According to the instructions of the assay kits, NO and TNF-α contents in supernatant were measured. Nitric oxide kit (Jiangsu Beyotime institute of Biotechnology, Jiangsu, China) based on the classical Griess reaction was used to determine the NO content and ELISA kit (eBioscience, San Diego, CA, USA) to TNF-α.

#### Quantitative real-time reverse-transcription polymerase chain reaction (RT-PCR)

RAW 264.7 cells were cultured in 6-well plates with 2 ml of culture medium, and total RNA was isolated using the commercial mRNA purification kit (TaKaRa Biotechnology, Dalian, China) according to the manufacturer’s protocol. In addition, a spectrophotometer (Thermo Scientific, Delaware, USA) was applied to determine the concentrations and purities of total RNA. gDNA removal and inverse transcription reaction were accomplished using commercial reagent kit (TaKaRa Biotechnology, Dalian, China).

Real-Time PCR System (Applied Biosystems, Life Technologies) was used to perform the real-time PCR assay with SYBR Green qPCR reagent kit (TaKaRa Biotechnology, Dalian, China), and β-actin was used as a housekeeping gene to normalize the threshold cycle (Ct) values of target genes. The specific primers for mouse iNOS, IL-6, TNF-α, IL-1β and β-actin are as follows: iNOS, sense primer 5'-CAGCTGGGCTGTACAAACCTT-3', antisense primer 5'-CATTGGAAGTGAAGCGTTTCG-3'; IL-6, sense primer 5'-AATGATGGATGCTACCAAACTG-3', antisense primer 5'-GGACTCTGGCTTTGTCTTTCT-3'; TNF-α, sense primer 5'-TCACACTCAGATCATCTTCTC-3', antisense primer 5'-AGACTCCTCCCAGGTATATG-3'; IL-1β, sense primer 5'-TTTGAAGTTGACGGACCCC-3'; antisense primer 5'-GATGTGCTGCTGCGAGATT-3'; β-actin, sense primer 5'-TGTCCACCTTCCAGCAGATGT-3', antisense primer 5'-AGCTCAGTAACAGTCCGCCTAGA-3'.

#### Western blot analysis

For obtaining whole cell extracts, RAW 264.7 cells were planted into 6-well plates. After treated with indicated procedures, the cells were washed with ice-clod PBS three times before lysed with RIPA buffer (50 mM Tris-HCl, pH 7.4, 150 mM NaCl, 5 mM EDTA, 1% NP-40, 0.5% sodium deoxycholate, 0.1% SDS, 1% aprotinin, 50 mM NaF, 0.1 mM Na_3_VO_4_) supplemented with protease and phosphatase inhibitor (Roche Molecular Biochemicals) which is before kept on ice for 30 min. The cell lysates were centrifuged (16,000×g) at 4°C for 20min. 40μg of total protein was separated on 10% sodium dodecyl sulfate polyacrylamide gel electrophoresis and transferred to PDVF membranes. The membranes were blocked with 3% BSA for 1.5 h at room temperature, and then probed with primary antibodies at 4°C overnight. The following antibodies were used for western blot: Phospho-NF-κB p65 (Ser536) (93H1), Phospho-IκBα (Ser32) (14D4), Phospho-p38 MAPK (Thr180/Tyr182) (12F8), Phospho-SAPK/JNK (98F2), Phospho-p44/42 MAPK (Erk1/2) (Thr202/Tyr204), c-Jun, p44/42 MAPK (Erk1/2), JUK, iNOS, COX2 (Cell Signaling Technology), anti-NF-κB p65 antibody (Chemicon (Millipore)) and IκB-α antibody (Santa Cruz Biotechnology).

The membranes were incubated with horseradish peroxidase conjugated secondary antibody for 2 h at room temperature, ECL substrate (Bio-Rad Laboratories, Richmond, California, USA) was used to detect bands.

#### Statistical analysis

Data are presented as means ± standard error (Western blot analysis were repeated three independent experiments with the same result). All experiments were done at least three times. One-way analysis of variance was used to compare the differences of means for three or more groups, and student’s t-test was performed to determine the statistical significance between two groups. P values < 0.05 was considered as statistically significant.

## Results and Discussion

### The major ingredients on inflammatory pathways

**Fig 2:**
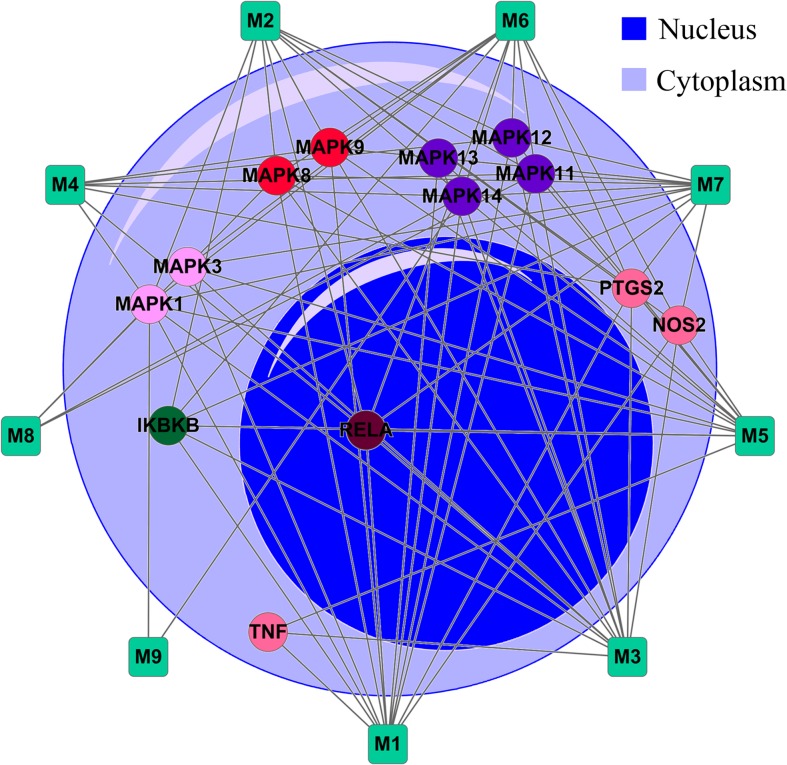
Drug-target (D-T) network. Circles and round rectangles correspond to the target proteins and drugs, respectively. Target nodes are colored according to their protein families. A link represents the interaction between a drug and a target node based on an in-house method (RF value ≥ 0.7 or SVM ≥ 0.8).

The relatively abundant components, chlorogenic acid, isochlorogenic acid A, isochlorogenic acid C and secoxyloganin from *Lonicera japonica Thunb*. Genipin, geniposide, gardenoside and geniposidic acid from *Gardenia jasminoides J*.*Ellis* and scopoletin, salicylic acid from *Artemisiae annuae L*. in RDN were applied for network analysis. As shown in **Fig 2**, salicylic acid was rejected due to the lack of interaction with selected targets. The rest 9 drugs and their targets generate a bipartite graph of D–T network through 85 edges. In this net, a large portion of proteins (8/13) connect with at least 7 drugs, and at least 2 proteins share 1 drug. Due to the multi-target drugs that modulate these essential proteins on parallel pathways or similar functional pathways may produce enhanced effect [4,14], these results indicate that RDN may enhance the therapeutic efficacy by collective regulation of targets in the signaling networks. In addition, MAPK1 and MAPK14 are the hubs with the highest degree of nine, demonstrating that these proteins may act as the major therapeutic targets for RDN, whereas, the downstream targets of the inflammatory pathways have relative lower degrees (e.g. TNF-α with the degree of 3), indicating that RDN cure inflammation mainly through the modulation of these signaling pathways.

### Experimental validation

#### The anti-inflammatory efficacy of RDN in LPS stimulated RAW 264.7 cells

In order to validate these results predicted by pathway-based method, we firstly evaluated the anti-inflammatory effect of RDN in LPS stimulated RAW 264.7 cells.

#### The viability of RAW 264.7 cells treated by RDN

**Fig 3:**
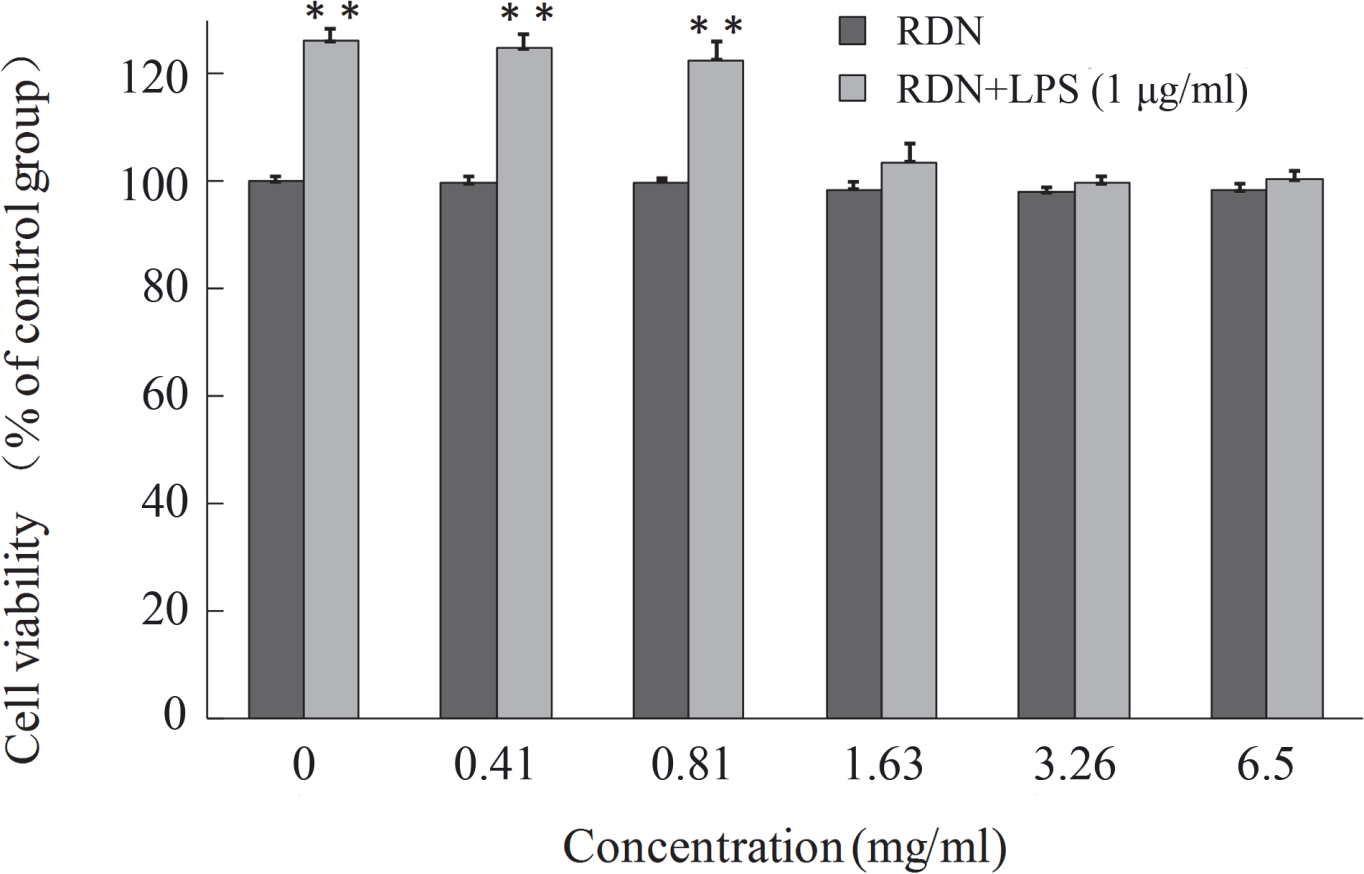
Cell viability of RAW 264.7 cells (4×105 cells/ml) in response to RDN and LPS. The viability of RAW 264.7 cells was determined by CCK-8 assay after incubated with indicated concentrations of RDN for 24 h supplemented with or without 1 μg/ml of LPS. **p < 0.01 represents significant difference compared with the control group.

To investigate the effect of RDN on cell viability, RAW 264.7 cells were exposed to various concentrations of RDN for 24 h. As shown in **Fig 3**, the cell viability of control group (cultured in fresh medium with less than 0.1% DMSO without test samples and LPS) was regarded as 100%. At the dosage of 0.41 to 6.5 mg dried medicinal herbs per milliliter culture media of RDN has no significant toxic effects on RAW264.7 cells.

The viability of RAW 264.7 cells stimulated with LPS was markedly increased to 126.7% compared with the control group. On the contrary, when treated with test samples (at the dosage of 1.63 to 6.5 mg/ml), cells were came back to normal conditions. These indicate that this formula may be a potential anti-inflammatory drug.

#### RDN decreases NO production

**Fig 4:**
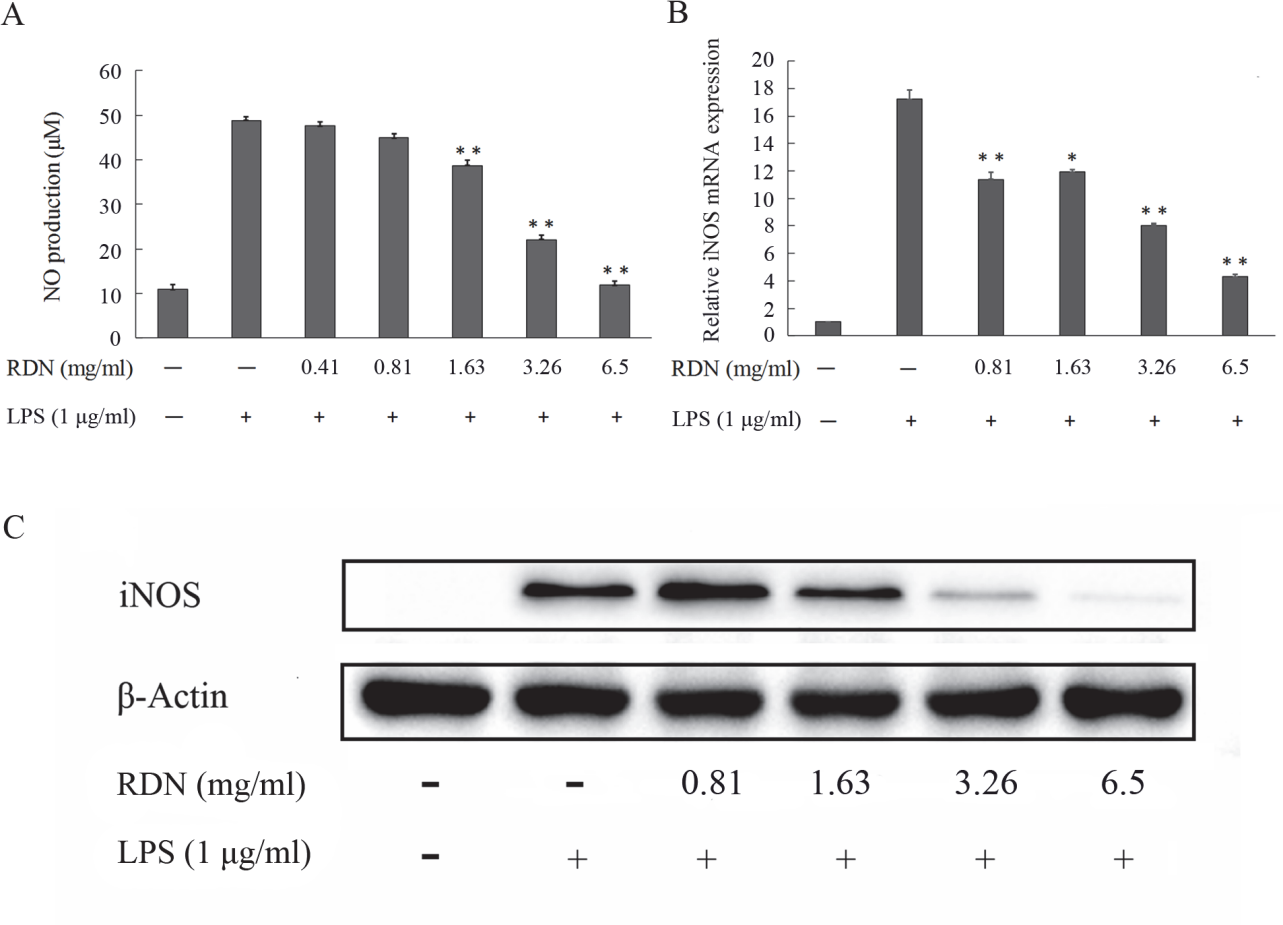
Effects of RDN on the production of NO, iNOS mRNA and protein in RAW 264.7 cells. Cells were pre-incubated with or without RDN at various concentrations for 2 h and then stimulated with LPS (1 μg/ml) for an indicated time. NO production (A) in culture medium was determined after cultured with LPS for 24 h, the gene expression of iNOS (B) was measured by quantitative real-time PCR after stimulated with LPS (1 μg/ml) for 8 h, and the protein level of iNOS (C) in whole cell extracts were determined after being stimulated for 18 h by western blot. Data are presented as mean ± standard error of three independent experiments in triplicate. *P < 0.05 and **P < 0.01 represent significant difference when compared with LPS group.

Since no evident cytotoxicity was observed when RAW 264.7 cells were cultured with the concentrations of RDN from 1.63 to 6.5 mg/ml. For RAW 264.7 cells, when exposed to LPS only, the NO levels increased from 10.9 to 48.81 μM, however, when treated with RDN from 1.63 to 6.5 mg/ml, the NO accumulation was significantly inhibited in a dose-dependent manner (**Fig 4A**). NO is considered as an important regulator of body homeostasis and its production can be used to evaluate the anti-inflammatory effects of a drug [24,25]. To testify why RDN could decrease NO production, the expressions of iNOS mRNA and protein were then determined by quantitative real-time PCR and western blot respectively. **Fig 4B** suggested that the expression of iNOS mRNA was observably reduced, meanwhile, the iNOS protein in the RDN and LPS treated cells was significantly decreased compared with the LPS-treated cells (**Fig 4C**). All these demonstrate that RDN acts on the signaling pathways, supporting the above *in slico* analysis.

#### RDN inhibits the mRNA expression of pro-inflammatory cytokines

**Fig 5:**
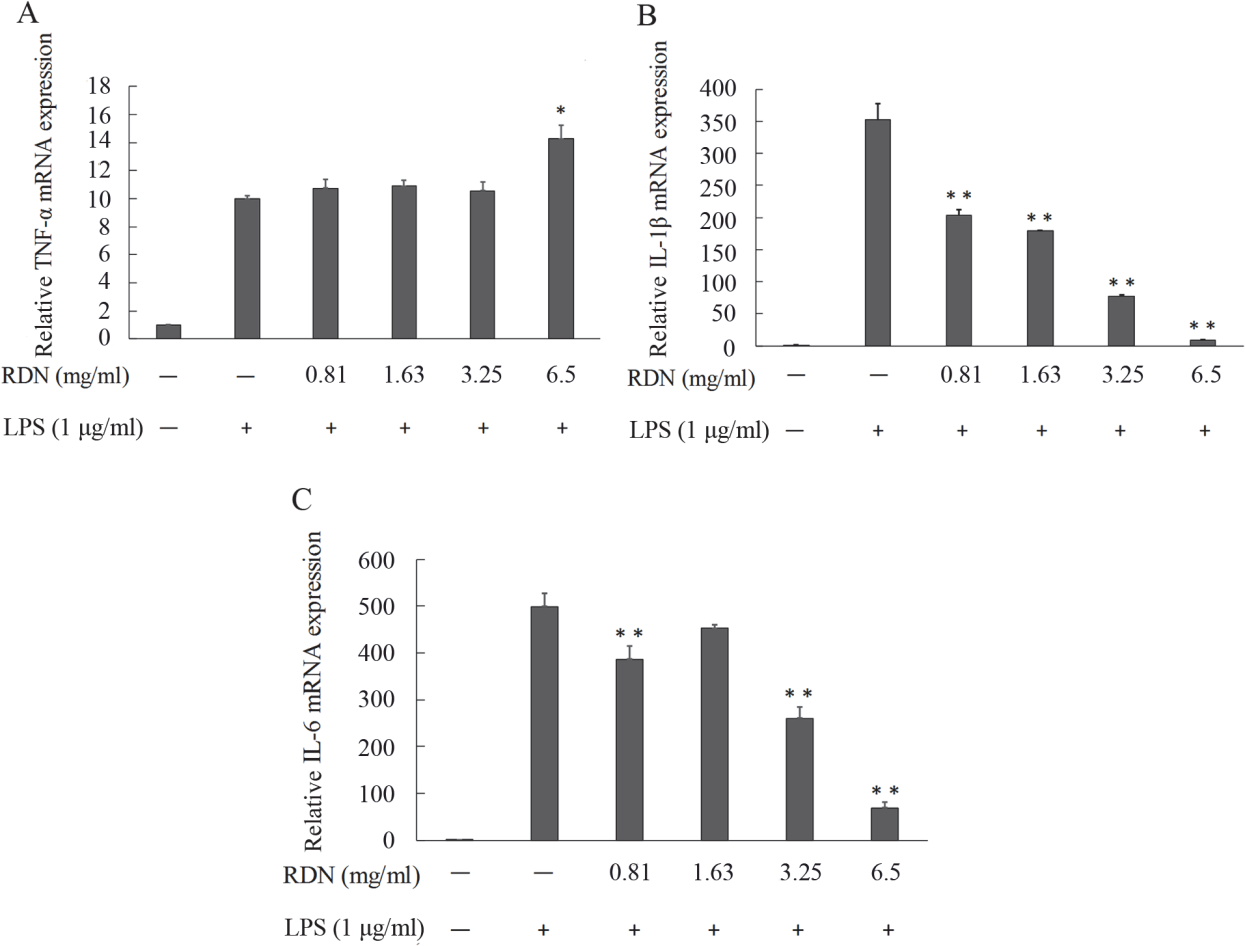
The mRNA expression of pro-inflammatory cytokines in RAW 264.7 cells. RAW 264.7 cells pretreated with 2-fold serial diluted RDN for 2 h were cultured in absence or presence of LPS (1 μg/ml) for 8 h. mRNA levels of TNF-α (A), IL-1β (B) and IL-6 (C) were quantified by quantitative real-time PCR. Data were presented as mean ± standard error of three independent experiments in triplicate. *P < 0.05 and **P < 0.01 represent significant difference compared with the cells that treated with LPS only.

The quantitative real-time PCR was carried out to investigate the effects of RDN on the mRNA expressions of IL-1β, TNF-α and IL-6. We found that the transcriptional levels of these pro-inflammatory cytokines genes were very low in RAW 264.7 cells without LPS. When incubated cells with 1 μg/ml LPS for 8 h, the expressions of these three genes were significantly increased. However, no substantial effects were observed on LPS-induced increased in the mRNA expression of TNF-α treated with RDN in our test (**Fig 5A**). On the contrary, when treated with various concentrations of RDN (ranging from 0.41 to 6.5 mg dried medicinal herbs per milliliter culture media), the mRNA levels of IL-1β (**Fig 5B**) and IL-6 (**Fig 5C**) were significantly decreased compared with the LPS-stimulated alone. In a word, these results prove that RDN exhibits good anti-inflammatory performances.

#### The integrative effect on NF-κB and MAPKs pathways

In the computational analysis, we found that RDN may systematically perturb the NF-κB and MAPKs pathways, which was partly validated by the following experiments.

#### The suppressive effect of RDN on NF-κB pathway

**Fig 6:**
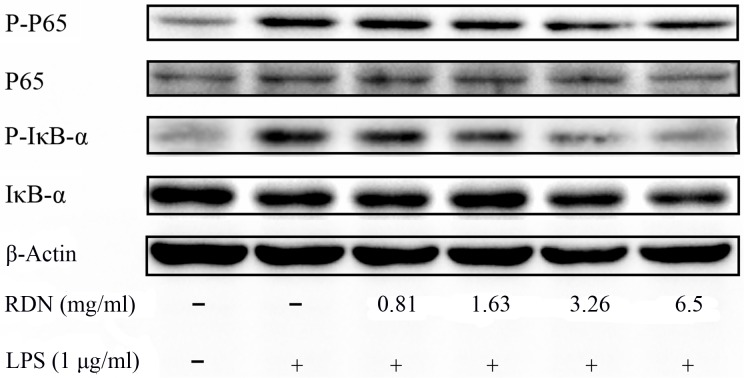
Effects of RDN on LPS-induced NF-κB activation in RAW 264.7 cells. Cells were pretreated with different doses of RDN for 2 h, followed by stimulated with LPS (1 μg/ml) for 30 min. The phosphorylated p65, IκB-α and total p65, IκB-α were immunoblotted with specific antibodies, with β-actin being used as control. Results were repeated through three independent experiments with the same tendency.

To validate whether the NF-κB pathway can be regulated by RDN, the phosphorylated p65, IκB-α and total p65, IκB-α were examined by western blot. Stimulated with LPS alone for 30 min increased the NF-κB p65 phosphorylation compared with control group. As expected, RDN inhibited the phosphorylated p65 in a dosage dependent manner. Moreover, the increased phosphorylation of IκB-α incubated with LPS alone was also decreased by addition of various concentrations of RDN (**Fig 6**). In the un-stimulated cells, NF-κB dimmers are sequestered in the cytoplasm by IκB inhibitory proteins. Once IκB proteins were phosphorylated by extracellular signals, NF-κB was released and then entered into nucleus to regulate specific gene expression [26]. Hence, RDN may block certain genes transcription partly by lowering the phosphorylation of IκB-α and the activation of NF-κB p65.

#### The modulation effect of RDN on MAPKs pathways

To evaluate the molecular mechanism of RDN on MAPKs modulation, RAW 264.7 cells were pretreated with indicated concentrations of RDN for 2 h, then 1 μg/ml LPS was added for an indicated time. Finally, phosphor-specific and non-phosphor-specific antibodies were used to probe the phosphorylated and non-phosphorylated p38, ERK1/2, and JNK respectively in total cell lysates.

Through CD14 and toll-like receptor, the administration of LPS to macrophages results in an activation of the mitogen-activated protein (MAP) kinase pathways [27–29], which in turn activates a variety of transcription factors such as NF-κB and AP-1, then finally regulates the production of pro-inflammatory cytokines as well as downstream signaling events that lead to inflammation. p38 MAPK is implicated in the regulation of leucocytes migration and accumulation, cytokines and pro-inflammatory mediators production [30]. For ERK1/2, the activated ERK1/2, involved in the regulation of meiosis, mitosis and postmitotic functions in differentiated cells [31], is important for T cell activation and differentiation [32]. With regard to JNK, it was discovered to phosphorylate the c-Jun, a major component of AP-1, leads to increase its transcriptional activity of many cytokine genes [31].

**Fig 7:**
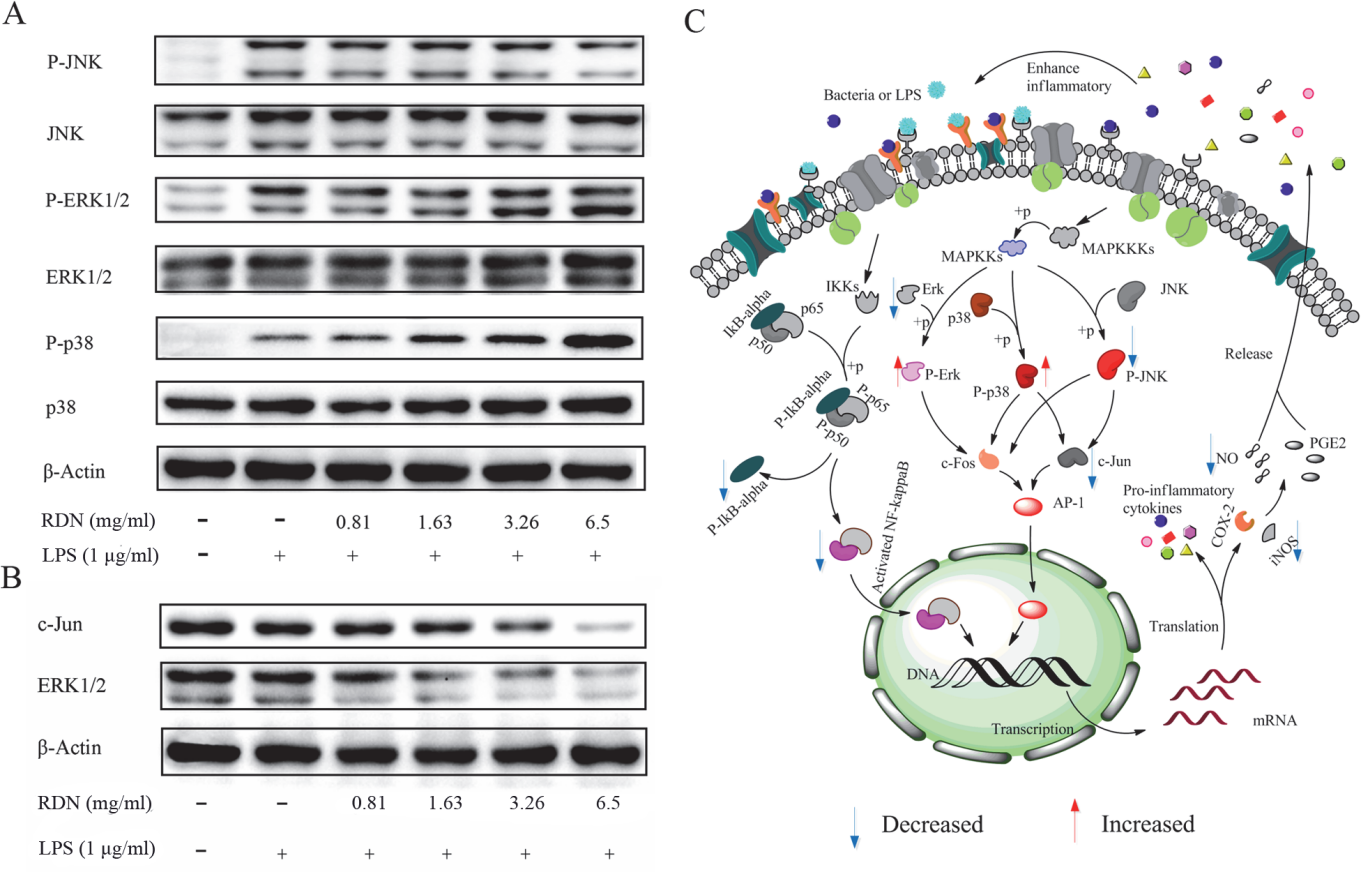
Effects of RDN on LPS-induced MAPKs activation in RAW 264.7 cells. Cells were pretreated with control solution or RDN for 2 h, followed by incubation with or without LPS (1 μg/ml) for a fixed time. Phosphorylated ERK1/2, p38 and JUK as well as non-phosphorylated proteins were detected after being incubated for 30 min (A). And after 18 h, the proteins of ERK1/2 and c-Jun regardless of phosphorylated or not were also examined (B). And (C) depicts the putative modulation pathways that respond to RDN therapeutic molecular mechanisms in LPS-stimulated RAW 264.6 cells, where blue downward arrows and red upward arrows represent decreased and increased tendency of a target respectively.

As shown in **Fig 7A**, stimulated with LPS for 30 min results in a significant increasing in the amount of phosphorylation of JNK, p38 and ERK1/2 compared with the control group. No changes in the total ERK, JNK and p38 kinase were observed in RAW 264.7 cells when treated with LPS or LPS and RDN for 30 min. The LPS-induced increasing of the activated form of JNK in RAW 264.7 cells was reduced in a dose-dependent manner by addition of gradient concentration of RDN. On the contrary, the activation of p38 MAP kinase was significantly increased in RAW 264.7 cells after being incubated with LPS and RDN, but the production of TNF-α was unchanged (data are not shown) and the mRNA of IL-1β and IL-6 were markedly decreased compared with LPS stimulated cells (Fig 5B and 5C), which is in coincidence with a previous report [27]. For the increased activation of ERK1/2 after being incubated with LPS and RDN for 30 min, it may enhance the cell-mediated immunity to remove foreign substances which give rise to inflammation in the early stages. Interestingly, in a dosage-dependent manner, total protein expression levels of ERK1/2 and c-Jun were decreased in cells after treated with RDN for 18 h (**Fig 7B**). The regulation action of RDN in LPS-stimulated RAW 264.7 cells may be associated with the earlier and persistent effects.

All these results above suggest that RDN may comprehensively modify the NF-κB and MAPKs pathways to resist inflammation (**Fig 7C**).

#### The joint-action effects of three major anti-inflammatory components

Initial experiment by measuring NO production in LPS-stimulated RAW 264.7 cells has ready demonstrated that genipin and scopoletin can significantly reduce the NO production (**Table 1**). In addition, chlorogenic acid is the most abundant ingredient extracted from *Lonicera japonica Thunb* in RDN and can significantly depress COX-2 expression in LPS-stimulated inflammatory model [33]. More importantly, these three compounds can be combined together to produce expected therapeutic properties by our PreDC computation [34]. Thus, presently, chlorogenic acid (C), genipin (G) and scopoletin (S) were chosen as the chemical markers to investigate the potential joint-action effects in anti-inflammation.

**Fig 8:**
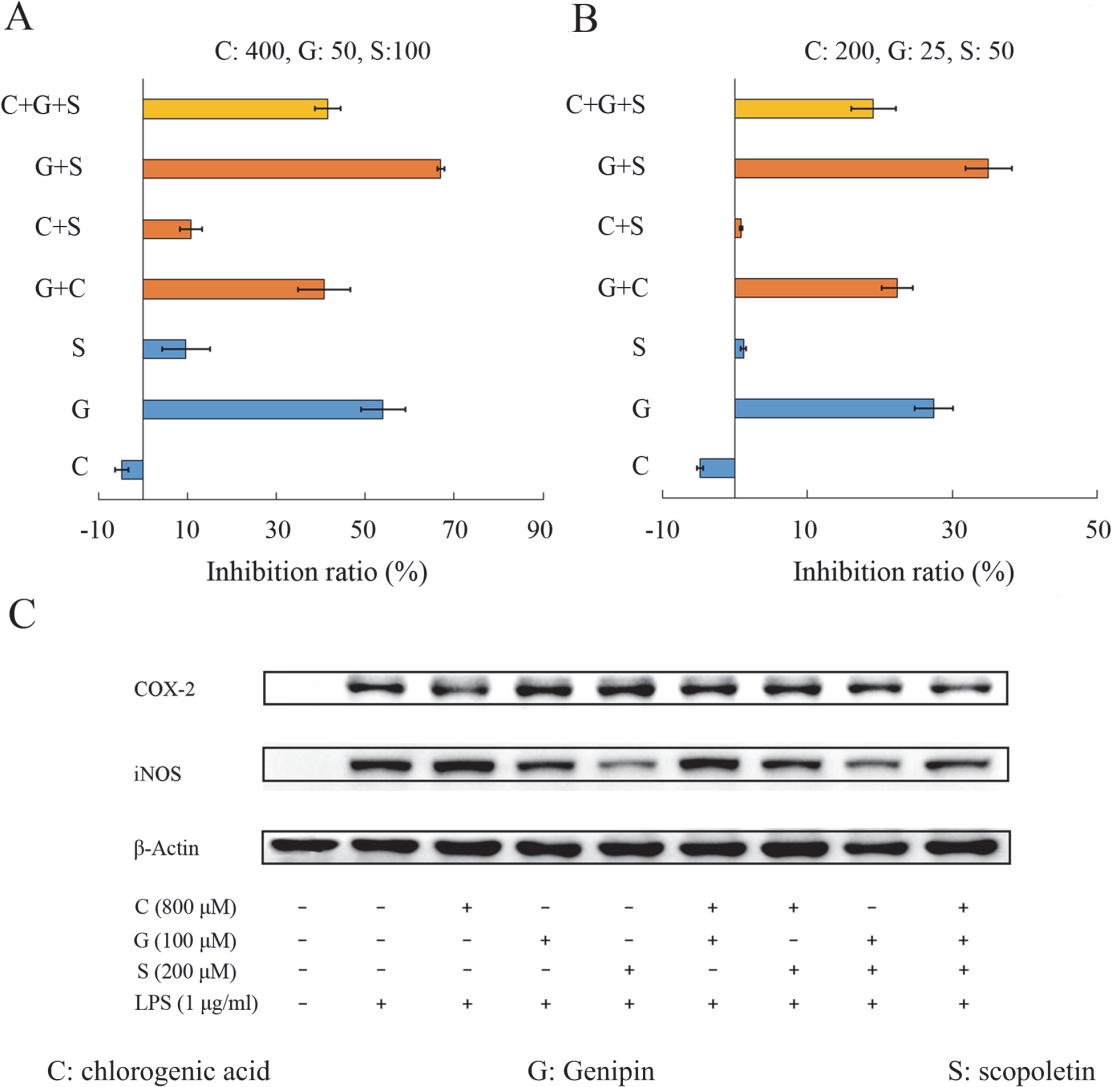
The joint-action effects of C, G and S. Cells were pre-incubated for 2 h with or without test agents, and then incubated with LPS (1 μg/ml) for an indicated time. The joint-action effect on NO production (A and B) was examined after stimulated with LPS for 24 h, and 18 h for COX-2 and iNOS (C) expression, respectively.

To study the joint-action inhibitory efficacy on NO production, the effects of the mixtures on NO production levels in LPS-stimulated RAW 264.7 cells were determined using a fixed ratio design. As depicted in **Fig 8A**, among the three compounds, G was the most effective element in inhibition of NO production at the concentration of 50 μM with an inhibition ratio of 54%, followed by 9.7% for S at the dosage of 100 μM, and C seemed to increase the NO production with a negative inhibition ratio. The binary and ternary mixtures of these three compounds exhibited different levels of depression effects. The inhibition ratio of NO production was 66.9% when combined G and S at the total concentration of 150 μM, while an inhibition of 54% and 9.7% were observed for G and S at the dosage of 50 and 100 μM, respectively. And when combined C and G or S at the total concentration of 450 or 500 μM, the inhibition ratio was 40.8% and 10.7%, respectively. The same tendency was also observed at a low concentration (**Fig 8B**). Synchronously, the protein of COX-2 and iNOS were examined by western blot analysis (as shown in **Fig 8C**). In the monotherapy treatment, C but not G or S triggers the degradation of COX-2 expression, and for iNOS, G and S revealed the blocked results, whereas C was not an inhibitor for iNOS expression. The result was a balance system when combined these three constituents in RDN to block both COX-2 and iNOS expressions.

Chlorogenic acid attenuates the activation of NF-κB and JNK/AP-1 signaling pathways to decrease LPS-induced up-regulation of COX-2 but not ERK1/2 and p38 pathways [33]. Genipin is found to prevent the activation of MAPKs and NF-κB [35]. And scopoletin shows a concentration-dependent inhibition of NF-κB pathway and the phosphorylation of MAPKs [36]. Through the mutual accentuation, counteraction and antagonism effects on MAPKs and NF-κB pathways, chlorogenic acid, genipin and scopoletin systematically reduce both the iNOS and COX-2 expressions as well as the NO production.

## Conclusions

In this work, based on systems pharmacological method, we proposed a strategy which focuses on pathways to deeply explore the molecular pharmacology of TCM, which, we hope, may in turn advance the drug discovery and development. As an example, theoretically and experimentally, we analyzed the anti-inflammatory effects of RDN and derived the target network at signaling pathways level (**Fig 7C**). This method provides a systematic understanding of the mechanisms of herb medicines acting on a particular disease-related pathways, and in this way, accelerating the drug discovery process.
